# Supplementary material for: Altered Cortical Palmitoylation Induces Widespread Molecular Disturbances in Parkinson’s Disease
Source: Int J Mol Sci. 2022 Nov 14;23(22):14018. doi: 10.3390/ijms232214018 (PMC9696982; doi:10.3390/ijms232214018)
Supplement: Supplementary file 1 [file ijms-23-14018-s001.zip › Supplemantary Figure S1.pdf]

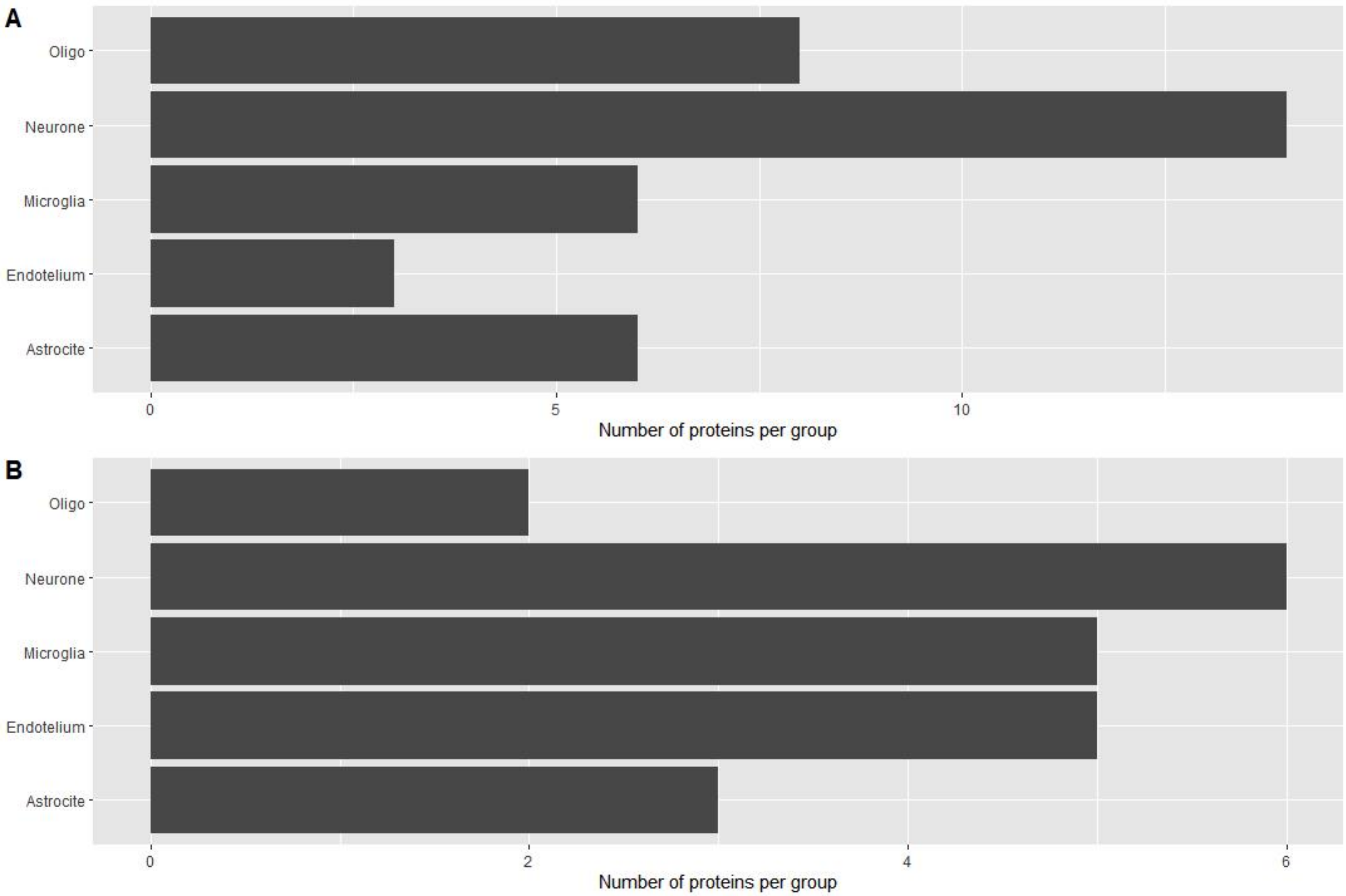

Supplementary Figure S1. Prediction of localization in cell types of proteins with altered levels of palmitoylation in the cortex of PD patients regarding controls. (A) Barplot showing the number of differential palmitoylated proteins (DPPs) located in different cell types of the brain. (B) Barplot showing the number of qualitative palmitoylated proteins (QPPs) located in different cell types of the brain.
