# Supplementary material for: Altered Cortical Palmitoylation Induces Widespread Molecular Disturbances in Parkinson’s Disease
Source: Int J Mol Sci. 2022 Nov 14;23(22):14018. doi: 10.3390/ijms232214018 (PMC9696982; doi:10.3390/ijms232214018)
Supplement: Supplementary file 1 [file ijms-23-14018-s001.zip › Supplementary Table S1.pdf]

Parkinson’s disease patients

Group	Code	Case ID	Age	Braak score	LB Stage	NIA-R	Dementia	Other Pathological Observations
Control	WT1	01-31	81	III	0. No LB	CNM	no	Microscopic changes of early or mild AD (insufficient for diagnosis).
	WT4	02-24	87	III	0. No LB	CNM	no	Mild microscopic changes of AD (insufficient for diagnosis).
	WT5	02-27	86	I	0. No LB	CNM	no	Non-specific changes.
	WT7	08-40	76	I	0. No LB	CNM	no	Normal brain for age.
Parkinson's disease	PD9	12-22	83	IV	IV. Neocortical	Low	yes	Microscopic changes of AD (insufficient for diagnosis).<br>Cerebral white matter rarefaction.<br>Microscopic focus of cerebellar cortical sclerosis.
	PD10	12-29	86	III	IV. Neocortical	CNM	yes	Non-specific changes.
	PD11	12-42	69	II	IV. Neocortical	Low	yes	Microscopic changes of early or mild AD (insufficient for diagnosis).
	PD12	98-03	72	I	IV. Neocortical	CNM	yes	Small old cortical infarctions of left cerebral convexity.

Supplementary Table S1. Neurological and pathological characteristics of PD patients and controls included in the study. **Case ID**, subjects listed by year and number, e.g., 01-31 is the 31st autopsy done in 2001. **Braak score** is the Braak neurofibrillary stage (0- VI) as originally defined by Braak and Braak, Acta Neuropathol. 82:239-59, 1991. **LB stage** is Lewy body stage, defined by Beach *et al*., Acta Neuropathol. 117:613-634, 2009. **NIA-R** (NIA-Reagan) is based on consensus recommendations for postmortem diagnosis of AD (The National Institute on Aging and Reagan Institute Working Group on Diagnostic Criteria for the Neuropathological Assessment of Alzheimer’s Disease, Neurobiol. Aging 18: S1-S2, 1997). High, intermediate, and low NIA-R refer to the likelihood that dementia, if present, is due to AD histopathology; CNM, criteria not met, refers to cases in which dementia was not present as NIA-Reagan criteria were meant to only be applied to cases with dementia. **Dementia** (clinical history) observed in the patient yes / no. **Other pathological observations** in the clinical history not previously commented. AD: Alzheimer´s disease.
