# Supplementary material for: Altered Cortical Palmitoylation Induces Widespread Molecular Disturbances in Parkinson’s Disease
Source: Int J Mol Sci. 2022 Nov 14;23(22):14018. doi: 10.3390/ijms232214018 (PMC9696982; doi:10.3390/ijms232214018)
Supplement: Supplementary file 1 [file ijms-23-14018-s001.zip › Supplementary Table S7.pdf]

Overlap of differential palmitoylated proteins (DPPs) with PD-related proteins´ interactomes		
**DPPs matching *SNCA*, *LRRK2* and *DJ-1* interactomes**	**DPPs matching *GBA*, *UCHL1* and PINK*1* interactomes**	**DPPs matching *SNCA*, *LRRK2*, *DJ-1 GBA*, *UCHL1,* and PINK*1* interactomes**
**DPPs / *SNCA***	**DPPs / GBA**	**DPPs / *LRRK2***
DSP	OGT	YWHAH
**DDPs / *LRRK2***	**Shared**	**DPPs / *GBA***
OPA1	FGG	OGT
YWHAH	FGB	**DPPs / SNCA / *LRRK2***
**DPPs / *DJ-1***	DHX9	HPRT1
IMPDH2	C3	MAPT
TXN	HSPA2	**DPPs / SNCA / *LRRK2 / DJ-1***
**DPPs / *SNCA / LRRK2***	TT	PDRX3
HPRT1	UBE2O	**DPPs / *GBA / UCHL1 / PINK1***
MAPT	IMPDH2	FGG
**DPPs / *LRRK2 / DJ-1***	OPA1	FGB
TKT	DDX17	DHX9
CFL1	PCYOX1	C3
DBN1	CFL1	HSPA2
**Shared**	KHSRP	UBE2O
PRDX3	DSP	DDX17
	DBN1	PCYOX1
	TXN	KHSRP
	ATP6V1A	ATP6V1A
		**DPPs / *SNCA / GBA / UCHL1 / PINK1***
		DSP
		**DPPs / *LRRK2 / GBA / UCHL1 / PINK1***
		OPA1
		**DPPs / *DJ-1 / GBA / UCHL1 / PINK1***
		IMPDH2
		TXN
		**DPPs / *LRRK2 / DJ-1 / GBA / UCHL1 / PINK1***
		TKT
		CFL1
		DBN1

Supplementary Table S7. Differential palmitoylated proteins (DPPs) in cerebral cortex from Parkinson' disease patients that interact with proteins encoded by relevant PARK genes (*SNCA*, *LRRK2*, *DJ-1 GBA*, *UCHL1,* and PINK*1*).
