# Supplementary material for: Altered Cortical Palmitoylation Induces Widespread Molecular Disturbances in Parkinson’s Disease
Source: Int J Mol Sci. 2022 Nov 14;23(22):14018. doi: 10.3390/ijms232214018 (PMC9696982; doi:10.3390/ijms232214018)
Supplement: Supplementary file 1 [file ijms-23-14018-s001.zip › Supplementaty Table S8.pdf]

Predicted cellular location of differential and qualitative proteins with altered palmitoylation in cerebral cortex from Parkinson's disease patients									
**Differential palmitoylated proteins (DPPs)**					**Qualitative palmitoylated proteins (QPPs)**				
Astrocites	***Neurons***	***Oligodendrocites***	***Microglia***	***Endotelium***	***Astrocites***	***Neurons***	***Oligodendrocites***	***Microglia***	***Endotelium***
L2HGDH	UBE2O	DNM3	C3	PLP2	FLNC	RGS7BP	DUSP15	DCXR	CSRP2
FGA	TUBB3	ENDOD1	FSCN1	CRIP2	CSRP2	SYT17	PPM1E	NMT1	TSPAN5
CSRP1	PDXP	TUBB4A	PTGDS	RAP1B	GNAO1	MECP2	HISTH2BC	CSTB	ZDHHC20
HSPA2	TUBB2A	MAPT	PIP4K2A			PPM1E	RFTN1	EPDR1	PTGFRN
NEBL	FXYD7	ANKS1B	HPRT1			TANC2		RFTN1	MYLK
CASKIN1	RGS17	CSRP1	RAP1B			LPCAT4			
	DSP	GPR37							
	DBN1	IGSF8							
	CAMK2B								
	MAPT								
	SNAP25								
	C3								
	IGSF8								
	OGT								

Supplementary Table S8. Predicted cellular location of differential and qualitative proteins with altered palmitoylation in cerebral cortex from Parkinson's disease patients.
